# Novel Subclone of Carbapenem-Resistant *Klebsiella pneumoniae* Sequence Type 11 with Enhanced Virulence and Transmissibility, China

**DOI:** 10.3201/eid2602.190594

**Published:** 2020-02

**Authors:** Kai Zhou, Tingting Xiao, Sophia David, Qin Wang, Yanzi Zhou, Lihua Guo, David Aanensen, Kathryn E. Holt, Nicholas R. Thomson, Hajo Grundmann, Ping Shen, Yonghong Xiao

**Affiliations:** First Affiliated Hospital of Southern University of Science and Technology (Shenzhen People’s Hospital);; Shenzhen, China (K. Zhou);; The Second Clinical Medical College of Jinan University, Shenzhen (K. Zhou);; Zhejiang University, Hangzhou, China (T. Xiao, Q. Wang, Y. Zhou, L. Guo, P. Shen, Y. Xiao);; Centre for Genomic Pathogen Surveillance, Cambridge, UK (S. David, D. Aanensen);; University of Melbourne, Melbourne, Victoria, Australia (K.E. Holt);; London School of Hygiene and Tropical Medicine, London, UK (K.E. Holt, N.R. Thomson);; Wellcome Trust Sanger Centre, Cambridge (N.R. Thomson);; University of Freiburg, Freiburg, Germany (H. Grundmann)

**Keywords:** Klebsiella pneumoniae, ST11, carbapenem resistance, subclonal shift, recombination, virulence, bacteria, bacterial infections, antimicrobial resistance, China

## Abstract

We aimed to clarify the epidemiologic and clinical importance of evolutionary events that occurred in carbapenem-resistant *Klebsiella pneumoniae* (CRKP). We collected 203 CRKP causing bloodstream infections in a tertiary hospital in China during 2013–2017. We detected a subclonal shift in the dominant clone sequence type (ST) 11 CRKP in which the previously prevalent capsular loci (KL) 47 had been replaced by KL64 since 2016. Patients infected with ST11-KL64 CRKP had a significantly higher 30-day mortality rate than other CRKP-infected patients. Enhanced virulence was further evidenced by phenotypic tests. Phylogenetic reconstruction demonstrated that ST11-KL64 is derived from an ST11-KL47–like ancestor through recombination. We identified a pLVPK-like virulence plasmid carrying *rmpA* and *peg-344* in ST11-KL64 exclusively from 2016 onward. The pLVPK-like–positive ST11-KL64 isolates exhibited enhanced environmental survival. Retrospective screening of a national collection identified ST11-KL64 in multiple regions. Targeted surveillance of this high-risk CRKP clone is urgently needed.

The global dissemination of carbapenem-resistant *Enterobacteriaceae* (CRE) has become an urgent public health concern (*1*,*2*). In 2016, the World Health Organization included CRE in a list of antimicrobial-resistant priority pathogens on which to concentrate future drug development strategies. Of note, carbapenem-resistant *Klebsiella pneumoniae* (CRKP) account for 60%–90% of clinical CRE infections in the United States, Europe, and China (*1*–*3*), resulting in an increased mortality rate of up to 40%–50% in nosocomial settings (*4*).

The dissemination of CRKP is mostly clonal, and the population structure is geographically specific. Since its emergence during the early to mid-2000s, sequence type (ST) 258 has become the most prevalent CRKP clone in North America, Latin America, and Europe (*5*). However, in Asia, especially China, ST11 is the predominant clone, accounting for up to 60% of CRKP (*3*). ST11 is a single-locus (*tonB*) variant of ST258, and both types belong to the clonal group 258. A recombination event is thought to have occurred between a recipient ST11 and a donor ST442-like strain, giving rise to ST258 during 1985–1997 (*6**,**7*). A phylogenomic study revealed that the ST258 population consists of >2 clades, resulting from an ≈215-kb recombination event that includes the capsule polysaccharide (*cps*) synthesis locus (*6*). The genetic differences generated by the resulting capsular switch are supposed to be primarily responsible for the ST258 diversification (*8*). Likewise, a segregation was identified in the ST11 population, resulting in >3 clades with different capsular loci (KL) (*9**–**11*). These studies consistently indicate that *cps* is a recombination hotspot in *K. pneumoniae*. However, the K-type distribution within ST11 in clinical settings is unclear. More important, the biological, epidemiologic, and clinical importance of capsular switches in ST11 remains poorly understood.

Of greater concern, a carbapenem-resistant hypervirulent *K. pneumoniae* ST11 outbreak clone was recently reported in eastern China (*12*). The outbreak strain was KL47 and hypermucoid and harbored a virulence plasmid carrying *rmpA2* and the aerobactin synthesis locus. Loss of the plasmid substantially alleviated virulence in a *Galleria mellonella* moth model. This finding indicates a worrying convergence of carbapenem resistance and hypervirulence in an already epidemic lineage of *K. pneumoniae*. Although incidence of carbapenem-resistant hypervirulent *K. pneumoniae* has remained low (*13*–*15*), understanding how this lineage emerged and evolved is crucial in controlling its further dissemination.

In this study, we measured the occurrence and clinical outcomes of bloodstream infections (BSI) caused by CRKP in a tertiary hospital in China during 2013–2017. We characterized the genomic alterations in the dominant ST11 population and ascertained associated changes in phenotype and pathogenicity traits.

## Materials and Methods

### Setting and Study Design

We performed a retrospective study in a 2,500-bed tertiary care hospital in China during January 2013–June 2017. We reviewed medical records of any patient with a blood culture positive for *K. pneumoniae* and a clinical course consistent with bacteremia (upon notification of the patient). Patients <16 years of age were excluded. If 1 patient had >1 episode of BSI caused by *K. pneumoniae* (BSI-KP), we included only the first episode. This study was approved by the institutional review board of the First Affiliated Hospital of Zhejiang University in China (approval no. 2017–442). Definitions of terms are detailed in Appendix 1.

### Microbiologic Assessment

We determined antimicrobial susceptibility by using the VITEK-II system (bioMérieux, https://www.biomerieux.com) and further confirmed by using the broth microdilution method. We defined carbapenem nonsusceptibility as MIC >2 mg/L for imipenem or meropenem or MIC >1 mg/L for ertapenem (*16*). We used multilocus sequence typing to identify ST11 (*17*). We estimated the pathogenicity of *K. pneumoniae* by testing *G. mellonella* infection, biofilm production, and neutrophil-killing resistance, as previously described (*18**–**20*) (Appendix 1). We evaluated the capacity of CRKP to survive on dry surfaces over time by using previously described methods (*21*), except that the stainless steel discs were replaced by Corning 24-well polystyrene microplates (Merck, https://www.sigmaaldrich.com/URL) and the concentration of bacteria was adjusted to 1 × 10^8^ CFU/mL.

### Whole-Genome Sequencing and Analyses

We sequenced 154 ST11 isolates by using an Illumina Hiseq2500 instrument (Illumina, https://www.illumina.com) with 2 × 125-bp paired-end libraries. We performed de novo assembly of the short-read data by using CLC Genomics Workbench version 10.0 (QIAGEN, https://www.qiagen.com) after quality trimming (Phred quality score >20). We performed long-read sequencing on 2 isolates (KP16932 and KP47434) by using the PacBio RSII platform (Pacific Biosciences, https://www.pacb.com) with a 10-kb library. A hybrid assembly of these 2 isolates was generated by using Unicycler 0.4.0 (*22*) with the short and long reads. We annotated the assemblies by using the RAST server (https://rast.nmpdr.org) and conducted multilocus sequence typing by using the CGE server (https://cge.cbs.dtu.dk). We performed plasmid analysis by Southern blotting and Blast (Appendix 1). We determined the presence or absence of resistance and virulence genes by using Ariba (*23*) with a custom gene database (https://figshare.com/s/94437a301288969109c2) and identified K-type by using Kleborate (https://github.com/katholt/Kleborate). We further detected mutations in *rmpA* and *rmpA2* by using blastn (https://blast.ncbi.nlm.nih.gov/Blast.cgi). We included genome assemblies of the isolates sequenced in this study and the 62 isolates published elsewhere (*10**–**12*,*24*–*26*) in the phylogenetic and temporal analysis (Appendix 2 Table 1).

### Statistical Analysis

Statistical analyses are described in Appendix 1. We conducted all statistical analyses by using SPSS Statistics 23 (IBM, https://www.ibm.com) and SAS 9.4 (SAS institute, https://www.sas.com).

## Results

### Capsular Switch in CRKP-ST11 over a 4-Year Period

We retrospectively screened 10,134 *K. pneumoniae* isolates to determine the proportion of BSI-CRKP. Of 705 nonrepetitive bloodstream isolates, 203 were CRKP. The proportion of *K. pneumoniae* and CRKP in BSIs increased from 17.1% to 45.5% during the study period (Table 1). ST11 was the predominant clone among BSI-CRKP isolates, accounting for 85.7% (n = 174); annual distribution was relatively stable (95.2%–91.1%).

**Table 1:** Prevalence trend of *Klebsiella pneumoniae* causing BSIs in a tertiary hospital, China, 2013–2017*

Isolate type	2013	2014	2015	2016	2017 (half year)	Score test for trend	p value†
Primary BSI/non-BSI isolates	123/610	133/635	201/723	149/687	99/398	1.3934	0.1635
CRKP/non-CRKP	21/102	35/98	53/148	49/100	45/54	6.0697	<0.001
CRKP-ST11/non-ST11 CRKP	20/1	28/7	46/7	39/10	41/4	−0.3116	0.7553
ST11-KL47/ST11-KL64	18/1	20/4	22/23	11/28	5/36	−7.5463	<0.001

*BSI, bloodstream infection; CRKP, carbapenem-resistant *Klebsiella pneumoniae*; ST, sequence type.  †Calculated by using Chochran–Armitage trend test.

Five KLs were detected in the BSI-CRKP-ST11 population: KL47 (n = 76), KL64 (n = 92), KL31 (n = 3), KL103 (n = 2), and KL105 (n = 1). The ratio of ST11-KL47 to CRKP-ST11 dropped from 90% (18/20) in 2013 to 12.2% (5/41) in 2017, whereas that of ST11-KL64 increased from 4.6% (1/20) in 2013 to 87.8% (36/41) in 2017. Thus, the ratio of ST11-KL47 to ST11-KL64 decreased substantially in the study period (Table 1), suggesting a KL shift among the CRKP-ST11 population over the 4-year period.

### ST11-KL64 Infections as Cause of Higher 30-Day Mortality

**Figure 1:**
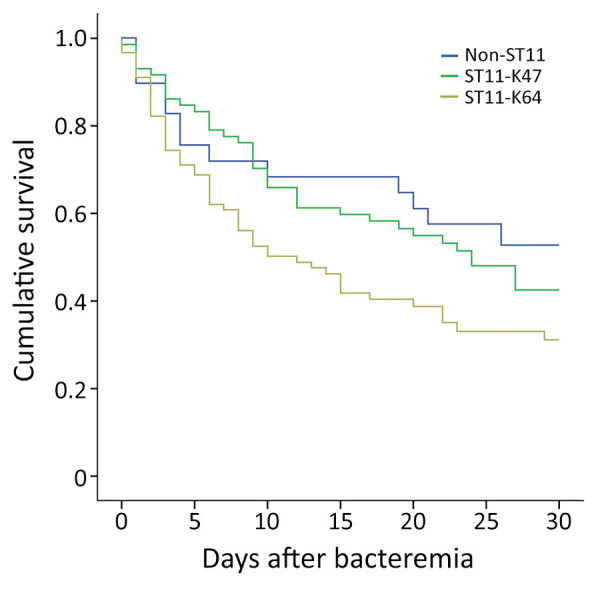
Kaplan–Meier survival estimates for patients with bloodstream infections caused by ST11-KL47, ST11-KL64, and non-ST11 CRKP, China, 2013–2017. A significant difference was found in the 30-day mortality among the 3 groups (p = 0.039). ST11-KL64–infected patients showed significantly higher overall 30-day mortality than ST11-KL47–infected patients (62.2% vs. 52.8%; p = 0.039) and non-ST11 CRKP–infected patients (62.2% vs. 44.8%; p = 0.05). No significant difference in 30-day mortality was found between patients infected with ST11-KL47 and non-ST11 CRKP (52.8% vs. 44.8%, p = 0.529). CRKP, carbapenem-resistant *Klebsiella pneumoniae*; KL, capsular loci; ST, sequence type.

To evaluate the clinical importance of ST11-KL47 and ST11-KL64, we analyzed 162 ST11-infected patients with complete clinical data, 72 patients with ST11-KL47 and 90 with ST11-KL64 (Appendix 2 Table 2); 4 ST11-KL47–infected and 2 ST11-KL64–infected outpatients were excluded. ST11-KL47 patients had a significantly longer stay than did ST11-KL64 patients, with respect to both the total hospital stay (p = 0.001) and hospital stay before the BSI onset (p = 0.029). More ST11-KL47–infected patients acquired lung infections and received invasive procedures, devices, or both before and after BSI; they also had received hemodialysis and chemotherapy or radiotherapy within 30 days before BSI. However, the Charlson comorbidity score was identical for patients of both groups. Patients infected with ST11-KL64 showed significantly higher overall 30-day mortality than those with ST11-KL47 (62.2% vs. 52.8%; 2 = 4.252; p = 0.039) (Figure 1).

We further included 29 patients infected with non-ST11 CRKP in the analysis to evaluate whether CRKP-ST11 caused higher mortality than non-ST11 CRKP. We found no significant differences in 30-day mortality between patients infected with CRKP-ST11 and those with non-ST11 CRKP (57.1% vs. 44.8%; χ^2^ = 0.833; p = 0.176). Cox regression multivariate analysis revealed 3 factors independently associated with a higher risk for ST11-caused mortality: lower platelet at time of BSI, Acute Physiology and Chronic Health Evaluation (APACHE II) score, and tigecycline as the empirical therapy (Table 2; Appendix 2 Table 3). We also found no significant difference in 30-day mortality between patients infected with ST11-KL47 and those with non-ST11 CRKP (52.8% vs. 44.8%; 2 = 0.395; p = 0.529). However, the ST11-KL64–infected patients showed significantly higher 30-day mortality than those with non-ST11 CRKP (62.2% vs. 44.8%; 2 = 3.771; p = 0.05).

**Table 2:** Cox regression of multivariable analysis of risk factors for 30-day mortality in 191 BSI patients infected with carbapenem**-**resistant *Klebsiella pneumoniae*, China, 2013–2017*

Variable	**p value†**	**OR (95% CI)**
Platelets at time of BSI	0.001	0.996 (0.994–0.998)
APACHE II score at time of BSI	0.012	1.041 (1.009–1.074
Tigecycline as empirical therapy	0.003	1.920 (1.257–2.935)

*APACHE, Acute Physiology and Chronic Health Evaluation; BSI, bloodstream infection; OR, odds ratio. †Calculated by using Cox regression.

### Recombination-Mediated Evolutionary Diversification in CRKP-ST11

**Figure 2:**
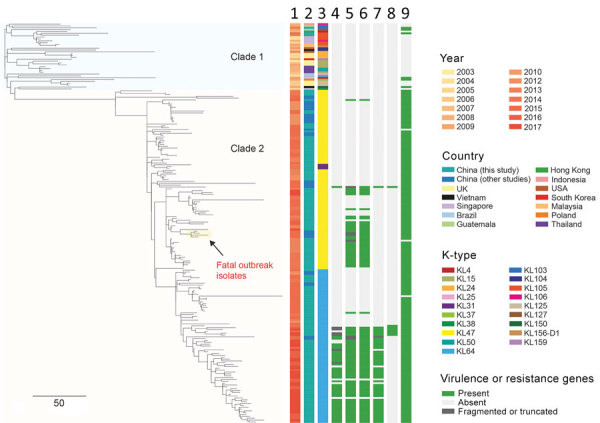
Phylogenetic analysis of 216 CRKP ST11 isolates, China, 2013–2017, including 154 CRKP isolates collected during 2012–2017 in study of bloodstream infections in a tertiary hospital and 62 isolates that were sequenced in previous studies (Appendix 2 Table 1). The phylogenetic tree was obtained by mapping all sequence reads to the hybrid assembly of KP47434 and removing the recombined regions from the alignment. The tree was rooted using ST1731 isolate EuSCAPE_ES29 (ERR1541319), which was included in this analysis but later removed from the tree (a tree including this outgroup is shown in Appendix 1 Figure 1). Five capsular types (KL31, KL47, KL64, KL103, and KL105) were detected in our ST11 collection, which are indicated in different colors as shown in the legend. Some of virulence genes detected are shown here. The *rmpA2* gene carried by KL64 isolates was frameshifted, namely rmpA2*. Aerobactin and salmochelin represent the *iucABCD-iutA* and *iroBCDN* gene clusters, respectively. The fatal outbreak clone reported in China recently (*12*) is highlighted on the tree. Lanes: 1, year; 2, country; 3, K-type; 4, *rmpA*; 5, *rmpA2*; 6, aerobactin; 7, *peg-344*; 8, salmochelin; 9, *bla*_KPC_*.* Scale bar indicates single-nucleotide polymorphisms. CRKP, carbapenem-resistant *Klebsiella pneumoniae;* KL, capsular loci; ST, sequence type.

We performed phylogenomic analysis to understand the evolutionary diversification in the CRKP-ST11 population. We included 154 newly sequenced genomes (excluding the remaining 20 isolates without *rmpA or rmpA2*); 62 previously published ST11 genomes from diverse origins; and an ST1731 genome (accession no. ERR1541319) as an outgroup. We identified 429 recombined regions, including 348 that were >1 kb. The length of sequence removed per isolate ranged from 505,312 to 1,276,214 bp (median 947,836 bp). The phylogenetic tree, which was rooted using the ST1731 outgroup isolate that was later removed (Appendix 1 Figure 1), showed division of ST11 isolates into 2 major clades (Figure 2). One clade consists of isolates of KL47, KL64, and KL31 exclusively obtained from China, whereas the second clade consists of isolates possessing diverse K-types from elsewhere. These findings suggest that KL47 and KL64 have emerged and undergone local expansion in China. 

Root-to-tip regression analysis of the 154 newly sequenced genomes demonstrated a correlation between the genetic distances and sampling dates (*R^2^* = 0.64) (Appendix 1 Figure 2). By using a Bayesian dating method implemented in BactDating (*27*), we found that KL64 isolates probably evolved from a KL47 ancestor around 2011 (Appendix 1 Figure 3). A high substitution rate also was found (15.3 single-nucleotide polymorphisms (SNPs)/genome/y, 95% CI 12.4–19.0 SNPs/genome/y).

The number of SNPs separating ST11-KL47 and ST11-KL64 isolates was 907–3,098 before and 30–220 after removal of recombination regions. This finding suggests that recombination largely contributed to the diversification of ST11-KL47 and ST11-KL64. Indeed, we detected 4 recombination events of >1 kb on the branch coinciding with the switch from KL47 to KL64 (regions with respect to the reference genome [isolate KP47434]: 307,448–322,057; 4,060,806–4,154,013; 4,173,036–4,186,742; and 4,197,111–4,217,597) (Appendix 1 Figure 4; Appendix 2 Table 4). Three of these events were localized around the *cps* region, suggesting that the capsule switch was likely the result of recombination. Another recombination event in the *cps* region also corresponds with the capsule switch from KL47 to KL31 (Appendix 1 Figure 4).

### Emergence of *rmpA*-*rmpA2*–Positive ST11-KL64 Isolates

Analysis of virulence genes showed that the 154 ST11 isolates possessed yersiniabactin genes (*ybtAEPQSTUX*, *irp1, irp2*, and *fyuA*) located on ICEKp3, and the core type III fimbrial cluster *mrkABCDF*, except for 4 ST11-KL64 isolates. Of 76 ST11-KL47 isolates, 29 (36.3%) carried an *rmpA2* gene, and only 2 were positive for the string test, suggesting that *rmpA2* was inactive in most isolates. *rmpA2*-positive ST11-KL47 isolates have been detected since 2013 and are interspersed among *rmpA2*-negative isolates in the phylogenetic tree (Figure 2). A frameshift *rmpA2* gene (rmpA2*) was identified in 48 of 92 ST11-KL64 isolates (52.2%). rmpA2*-positive ST11-KL64 isolates were first detected in 2015, and most of them are monophyletic (Figure 2). An *rmpA* gene was also found in 42 of the 48 rmpA2*-positive ST11-KL64 isolates, of which 6 had in-frame truncations resulting in 2 variants (555 bp and 624 bp) and 12 were positive for the string test. The *rmpA*-rmpA2*–positive ST11-KL64 isolates were detected from 2016 onwards. The prevalence trend of *rmpA/rmpA2*-positive isolates is accordant with that of each subclone (Appendix 1 Figure 5).

All rmpA/rmpA2*-positive isolates also carried aerobactin genes *iucABCD*-*iutA*, implying that they might co-locate on the same plasmid. A plasmidborne virulence factor *peg-344* was exclusively found in 45 of 48 rmpA2*-positive ST11-KL64 isolates. The salmochelin cluster *iroBCDN* was also detected in 5 *rmpA*-rmpA2*-positive and in 1 classic ST11-KL64 isolate (Figure 2).

### Diversity of Virulence Plasmids

We further analyzed the vectors of *rmpA*/*rmpA2* genes to understand how they were captured. The *rmpA*/*rmpA2* gene of both subclones was detected on plasmids by using southern blot (Appendix 1 Figure 6). Higher diversity of the virulence plasmids was found in ST11-KL64 through classifying the plasmids by size; 5 types were detected in ST11-KL47 with sizes ranging from 110 to 217 kb, and 13 types were in ST11-KL64, ranging from 110 to 230 kb (Appendix 2 Table 5). The *rmpA* and rmpA2* genes coexisted on the same plasmid in ST11-KL64.

Virulence plasmids detected in KP16932 (KL47) and KP47434 (KL64) were circularized to evaluate their structural variations. The *rmpA2* gene of KP16932 was carried by an IncFIB(K)-IncHI1B–type plasmid (pVir-KP16932) with a size of 177.8 kb, which is almost identical to a virulence plasmid pVir-CR-HvKP4 (MF437313) recently detected in a KL47 clone that caused a fatal outbreak in China (Appendix 1 Figure 7). The *rmpA* and rmpA2* genes of KP47434 existed in an IncFIB(K)-IncHI1B–type plasmid (pVir-KP47434) with a size of 201.8 kb, which shares a high homology with a virulence plasmid pVir-CR-HvKP267 (accession no. MG053312). Compared with pVir-KP47434, a 24-kb and an 18-kb region were absent in pVir-KP16932 (Appendix 1 Figure 7), which encodes genes involved in metabolic processes such as carbon utilization (OppA-B-F and DppC) (*28*) and virulence (H-NS protein) (*29*). The virulence plasmids carried by *rmpA2*-KL47 and rmpA/rpmA2*-KL64 isolates possessed the highly similar backbone sequences with the pVir-KP16932 and pVir-KP47434 plasmids, respectively, and the intra-subclonal variations were mainly caused by gain or loss of gene clusters involved in heavy metal resistance and mobile genetic elements (Appendix 1 Figure 8).

### Virulence Plasmids and Infections

To evaluate whether the acquisition of virulence plasmids carrying *rmpA/rmpA2* and aerobactin genes has an effect on clinical outcomes, we stratified the cohort described according to the existence of virulence plasmids in ST11-KL47 and ST11-KL64. No significant differences in mortality were evident between patients infected by ST11-KL64-pVir-KP47434–like or classical ST11-KL64 isolates (i.e., without virulence plasmids) (60.4% vs. 64.3%; p = 0.983) or those infected by ST11-KL47-pVir-KP16932 or classical ST11-KL47 isolates (51.7% vs. 53.5%; p = 0.931) (Appendix 2 Table 6, 7). However, the Charlson comorbidity scores for patients with ST11-KL47-pVir-KP16932–like infections (median 1 [range 0.5–2]) were significantly lower than scores for patients with ST11-KL47 infections (median 3 [range 1–4]; p = 0.003); similarly, scores for ST11-KL64-pVir-KP47434–like patients (median 1 [range 0–2]) were significantly lower than scores for ST11-KL64 patients (median 2 [range 1–3]; p = 0.003). These findings indicate that the virulence plasmids could promote infections in healthier patients.

### Various Resistomes in ST11-KL47 and ST11-KL64

The resistome of ST11-KL47 was different from that of ST11-KL64 (Appendix 1 Figure 9). Genes *floR*, *arr-3, dfrA27*, and *aac(6’)-Ib*-cr were exclusively detected in ST11-KL47, whereas *bla*_SHV-12_ and *dfrA14* were unique for ST11-KL64, suggesting the 2 subclones might have been selected for in different niches. For each subclone, the resistomes of *rmpA/rmpA2*-positive isolates were much more consistent than those of classical isolates (Appendix 1 Figure 9). This finding is consistent with the phylogeny and the fact that the *rmpA/rmpA2*-positive isolates were relatively more clonal.

### Enhanced Virulence in ST11-KL64

The *rmpA/rmpA2*-encoding virulence plasmids carried by each subclone shared an intrasubclonal similarity as described previously (i.e., the intrasubclonal variations were mainly caused by gain or loss of heavy metal resistance gene clusters and mobile genetic elements). ST11-KL64 isolates produced significantly more biofilm than ST11-KL47 isolates (optical density at 595 nm: 0.54 + SD 0.09 vs. 3.08 + SD 0.11; p<0.0001) (Appendix 1 Figure 10, panel A). We evaluated the virulence potential by using a human neutrophil assay. The ST11-KL64 isolates had an average survival of 91.2% after incubation with the human neutrophils for 60 min, which was significantly higher than that of the ST11-KL47 strains (65.8%; p = 0.0011) (Appendix 1 Figure 10, panel B). Compared with the ST11-KL64 isolates, the ST23-K1 isolates showed lower survival (70.3%) and the ST86-K2 isolates comparable survival (91.8%). The ST35 isolate had the lowest survival, 37.2%. 

We further estimated pathogenicity by infecting *G. mellonella* larvae with an inoculum of 1 × 10^6^ CFU. At 48 h postinfection, the 4 ST11-KL64 isolates (1 classic [isolate KP33068], 1 rmpA2*-positive [isolate KP33130], and 2 rmpA-rmpA2*-positive [isolates KP33229 and KP33367]) showed comparable virulence resulting in 10% survival, whereas survival was 40%–60% for the 4 ST11-KL47 isolates (2 classic [isolates KP9343 and KP29407] and 2 *rmpA2*-positive [isolates KP10042 and KP16932]). K1 survival 40% and K2 30%; survival of a classic CRKP isolate (ST35) reached 70% (Appendix 1 Figure 10, panel C).

To determine the underlying mechanisms of enhanced transmissibility obtained by *rmpA*-rmpA2*–positive ST11-KL64, we randomly selected 6 ST11-KL47 isolates (3 classic [KP8369, KP29407, and KP30412] and 3 *rmpA2*-positive [KP9343, KP10042, and KP16932]) and 6 ST11-KL64 isolates (2 classic [KP28367 and KP33068], 2 rmpA2*-positive [KP33130 and KP45812], and 2 rmpA-rmpA2*–positive [KP47434 and KP39615]) to evaluate the capacity of survival on a dry polystyrene surface. Only viable cells of 2 rmpA-rmpA2*-KL64 isolates were recovered after overnight drying; the average recovered loads were 90 + 31.09 CFU/mL and 115 + 20.62 CFU/mL. This finding suggests that the enhanced transmissibility of the newly emerged subclone was associated with enhanced environmental survival.

### National Prevalence of BSI-CRKP-ST11

To estimate the national prevalence of BSI-CRKP-ST11, we further retrospectively screened 1,098 clinical BSI-KP strains collected from 13 provinces in China during 2014–2016 (Table 3). In total, 46 of 83 CRKP strains were ST11; ST11-KL47 accounted for 80.4% and ST11-KL64 19.6%. The *rmpA2* gene was detected in 11 ST11-KL47 and 1 ST11-KL64 isolates, and 1 isolate of each subclone also co-harbored an *rmpA* gene. The *rmpA2*-positive isolates were detected from Anhui and Zhejiang provinces. Most (9/12) *rmpA2*-positive CRKP isolates appeared after 2015.

**Table 3:** Prevalence of CRKP-ST11 causing BSIs, China, 2014–2016*

Year	KP	CRKP	CRKP-ST11	CRKP-ST11-KL47, *rmpA/rmpA2-*positive	CRKP-ST11-KL64, *rmpA/rmpA2-*positive
2014	224	10	7	5 (1)	2 (0)
2015	345	31	19	16 (2)	3 (0)
2016	529	42	20	16 (8)	4 (1)
Total	1,098	83	46	37 (11)	9 (1)

*Isolates were collected as part of national surveillance for BSIs. BSI, bloodstream infection; CRKP, carbapenem-resistant *Klebsiella pneumoniae*; KL, capsular loci; KP, *Klebsiella* pneumoniae**; **ST, sequence type.

## Discussion

The global dissemination of CRKP poses a serious threat to public health. Control of CRKP in populations and healthcare networks thus becomes an urgent issue. However, efforts are often complicated by rapid evolution, especially among epidemic clones (e.g., ST11 and ST258). Therefore, tracking of evolutionary events and understanding their clinical importance are critical. We performed a comprehensive study to provide insight into the evolution of key virulence features of BSI-CRKP collected in China. We found 2 major KLs (KL47 and KL64) in the dominant clone BSI-CRKP-ST11. Capsule is known as an important immune-evasion molecule, and thus has become a popular target for vaccine design. Determining the prevalence of KLs is crucial for the development of capsule-based vaccines and phage-derived exopolysaccharide-depolymerase treatments, which are considered as novel approaches for the treatment of CRKP infections (*30*). Our study provides useful data for assisting the development of an immunotherapy for ST11-CRKP infections in China.

In this study, ST11 was partitioned into 2 clades, 1 consisting of ST11-KL47, ST11-KL64, and ST11-KL31, suggesting that these strains were diversified from a common ancestor. We found that sequences within the *cps* region of ST11-KL64 and ST11-KL31 were imported through recombination indicating the occurrence of capsule switching. By using a Bayesian approach, we found that ST11-KL64 might have emerged from an ST11-KL47–like ancestor in 2011. We further noted that the 2 ST11 subclones (ST11-KL47 and ST11-KL64) have spread nationally by interregional transmission. However, the lack of genome data about ST11-KL47 and ST11-KL64 from different origins hampers our understanding of spatial evolution at a global scale.

The notion of a rapid evolution of the ST11 population is supported by numerous *cps* variants (n = 19) and the very high evolutionary rate (15.3 SNPs/genome/year) detected in this study and others (*9*). Capsule switching has been suggested to be a common event across the wider *K. pneumoniae* population through large recombination events (*9**–**11*). We suppose that generating numerous descendants with various combinations of evolved chromosomes and capsules heavily contributes to the success of ST11 and its descendants (e.g., ST258). Of note, our study identified a clonal replacement in the CRKP-ST11 population over a 4-year period in a hospital. ST11-KL47, the dominant subclone before 2015, was progressively replaced by ST11-KL64. The population structure of ST11-K64 was monophyletic, implying that ST11-KL64 might have gained fitness and was ready to disseminate clonally like ST258. Also, KL64 is a more commonly observed capsule type than KL47, and has been detected in Brazil (*25*), Taiwan (*31*), Singapore (*32*), the United States, and Europe (*33*).

To understand the clinical importance of the clonal replacement that coincided with the capsular switch in the ST11 population, we analyzed the metadata of 162 infected patients. Patients infected by ST11-KL64 had significantly higher mortality rates than those infected by ST11-KL47 and non-ST11 CRKP. This finding is supported by the results of our phenotypic assays, which showed that ST11-KL64 was more virulent than ST11-KL47. Our findings suggest that the acquisition of virulence plasmids promotes the infection in healthier patients but is not associated with the increased mortality, indicating that other virulence factors might be involved. Capsular switching in the ST11 population might contribute to increased mortality. The capsule type is thought to be an important determinant for the pathogenicity of *K. pneumoniae*, like the notorious capsular serotypes K1 and K2. Similar associations are also identified in other species, such as *Acinetobacter baumannii* (*34*) and *Streptococcus* spp. (*35*,*36*). We also cannot exclude that the enhanced virulence and increased mortality might be associated with other chromosomal and plasmid variations.

We further noted that the newly emerged rmpA2*-positive ST11-KL64 isolates exclusively carried an *rmpA* gene. The presence of a truncated variant might confer an advantage through a more subtle activation of capsule expression in comparison to a strain with 2 fully functional variants present (*37*). In addition, the combination of RmpA and truncated RmpA2 was previously found predominantly in clinical isolates with a hypervirulent or hypermucoviscous phenotype (*38*). This finding is consistent with our study, given that rmpA-rmpA2*-ST11-KL64 isolates become the dominant clone after they emerged. We suppose that such combination might confer fitness to the population resulting in the replacement of *rmpA2*-ST11-KL47 by rmpA-rmpA2*-ST11-KL64. This supposition can be supported by the fact that rmpA-rmpA2*-ST11-KL64 isolates survive longer than ST11-KL47 in vitro, which largely facilitates a better dissemination of the population under nosocomial conditions. Besides the isolates found in Anhui and Zhejiang provinces in our study, 2 rmpA-rmpA2*-ST11-KL64 isolates have been detected in Shanghai and Henan provinces (*9*), suggesting that the newly emerged subclone has widely disseminated in China.

In summary, our study identified the emergence of a high-risk subclone of CRKP-ST11, resulting in enhanced virulence and transmissibility. The newly emerging descendant obtained enhanced environmental survival and poses a substantial threat to healthcare networks, suggesting the urgent need for tailor-made surveillance and stricter infection-control measures to prevent further dissemination in nosocomial settings.

Appendix 1 Additional information (background and figures) regarding emergence of a novel subclone of carbapenem-resistant *Klebsiella pneumoniae* ST11 with enhanced virulence and transmissibility, China.

Appendix 2 Additional information (tables) regarding emergence of a novel subclone of carbapenem-resistant *Klebsiella pneumoniae* ST11 with enhanced virulence and transmissibility, China.
