# Supplementary material for: Novel Subclone of Carbapenem-Resistant Klebsiella pneumoniae Sequence Type 11 with Enhanced Virulence and Transmissibility, China
Source: Emerg Infect Dis. 2020 Feb;26(2):289–97. doi: 10.3201/eid2602.190594 (PMC6986851; doi:10.3201/eid2602.190594)
Supplement: Appendix 1 — Additional information (background and figures) regarding emergence of a novel subclone of carbapenem-resistant Klebsiella pneumoniae ST11 with enhanced virulence and transmissibility, China. [file 19-0594-Techapp-s1.pdf]

Article DOI: https://doi.org/10.3201/eid2602.190594

Novel Subclone of Carbapenem-Resistant *Klebsiella pneumoniae* Sequence Type 11 with Enhanced Virulence and Transmissibility, China

Appendix

Definition of Terms

BSI was defined according to the Centers for Disease Control and Prevention guidelines (http://www.cdc.gov/nhsn/pdfs/pscmanual/17pscnosinfdef_current.pdf). BSI onset was defined as the collection date of the first positive blood culture. The probable infectious source was determined using CDC/National Healthcare Safety Network surveillance definitions; Primary BSI was recorded if no source was identified (http://www.cdc.gov/nhsn/PDFs/pscManual/17pscNosInfDef_current.pdf). Overall mortality included all causes of death during hospitalization. Nosocomial infection was defined according to the definitions proposed by Friedman et al. (*1*). Empirical therapy included all antimicrobials administered before definitive therapy. Definitive therapy referred to antimicrobial therapy administered after the susceptibility testing results were available and was defined as "appropriate" if an adequate course of at least one drug was administered to which the pathogen was fully susceptible or "inappropriate" if these criteria were not met (*2*).

Toxicity Test

The virulence of *K. pneumoniae* was estimated by infecting *Galleria mellonella* larvae as described previously (*3*). Briefly, overnight cultures of *K. pneumoniae* strains were washed with phosphate-buffered saline (PBS) and further adjusted with PBS to concentrations of 1×10^6 CFU/mL. The survival rate of the *G. mellonella* was recorded for 48 hours. All experiments were done in triplicate. One ST23-K1 and one ST86-K2 isolate were used as controls, and they both were identified as hypervirulent *K. pneumoniae* in a previous study (*4*).

Biofilm Production

The biofilm assay was performed as previously described (*5*). Briefly, 1×10^7 CFU/mL bacteria were inoculated into MH medium in polystyrene microtiter 96-well plates and incubated at 37°C for 24 h. Biofilm was stained using crystal violet, followed by eluting with 95% ethanol and quantifying by optical density (OD) measurement (OD595). Biofilm assays were triplicated and in each assay quantification was performed in eight wells.

Human Neutrophil Assay

The human neutrophil assay was performed as previously described (*6*). Briefly, neutrophils were purified from freshly drawn venous blood of six healthy human volunteers, who signed written consent before participation in the study. The neutrophils were re-suspended in PBS (pH = 7.4) with concentration adjusted to 1×10^7 cell/ml, and were used immediately. The bactericidal activity of neutrophils was measured by incubating 1×10^6 neutrophils with 4×10^7 CFU of opsonized *K pneumoniae* in 600 μl PBS at 37°C for 60 min. Then neutrophils were lysed with 0.1% Triton X-100 for 15 min on ice before diluting and plating on MH agar. The experiment was repeated twice. The bacterial survival index was expressed as the percentage of CFUs in the experimental test divided by CFUs in the control test.

Phylogenetic and Temporal Analysis

Sequence reads of 154 ST11 isolates sequenced in this study, and of 62 isolates published elsewhere (*7–12*) were mapped to the hybrid reference assembly, KP47434, using BWA (*13*). Single nucleotide polymorphisms (SNPs) were called using an in-house pipeline with SAMtools and bcftools, and a pseudo-genome alignment was generated. Recombined regions were detected using Gubbins (*14*). A phylogenetic tree was constructed using the SNPs outside of the recombination regions with RAxML using a GTR model and gamma correction (*15*). An ST258 genome was also included in a separate phylogenetic analysis, using the same methods, to establish the root of the ST11 tree. The phylogenetic tree and associated metadata were visualized together in Microreact (*16*) BactDating (*17*) was used to perform regression analysis of the root-to-tip genetic distance against sampling time for the 154 ST11 isolates sequenced in this study and to obtain a dated phylogeny based on a Bayesian approach. For both, the output from Gubbins was used as the input, together with the isolation dates. Default settings were used for the Bayesian function, except that the MCMC chain length was increased to 1 million. The MCMC convergence was tested by examining traces of the model parameters. The effective sample sizes of the model parameters were also determined using the R package, “coda,” and these were >200.

Screening of ST11-KL47 and ST11-KL64 Isolates

We retrospectively screened 1,098 clinical BSI-KP strains to detect ST11-KL47 and ST11-KL64 isolates. The strains were collected from 13 provinces in China during 2014–2016 in the frame of a national surveillance for BSIs. The 1,098 isolates were first screened by agar dilution to identify CRKP. The CRKP isolates were later screed through multiplex PCR with use of primers for *wzy*$_{KL47}$ (Fw: GGACGCACAGTTTCCCAATTCGC; Rev: GCCCACATGAACCCACTTGGCA) and for *wzy*$_{KL64}$ (Fw: TCAGTTCCGACCCTGATGCAGGTA; Rev: GCCAGAGCAACTATCATCCAAAGCCA)

according to a previous study (*18*). The positive isolates were further tested by MLST using the scheme of Institute Pasteur as described previously (*19*).

S1-PFGE and Southern Blotting

The plasmids and location of *rmpA* and *rmpA2* genes were determined by S1-nuclease digestion and pulsed-field gel electrophoresis (S1-PFGE), followed with southern blotting hybridizations as previously described (*20*). Briefly, bacterial DNA was prepared in agarose blocks and was digested by XbaI nuclease. The digested DNA fragments were separated using PFGE with conditions of 14 h at 6 V/cm, 14°C, with a pulse angle of 120° and a switch time from 1 to 10s. The separated DNA fragments were transferred to nylon membranes (Hybond N, Amersham, UK), hybridized with digoxigenin-labeled *rmpA* or *rmpA2* probes and detected using an NBT/BCIP color detection kit (Roche, Basel, Switzerland).

Detection of Virulence Plasmids

Detection of virulence plasmids was mainly dependent on BLASTn as described previously (*21*). The contigs of each genome were blasted against the reference plasmid and plotted by BLAST Ring Image Generator (BRIG). Two circularized virulence plasmids, pVir-KP16932 and pVir-KP47434, were used as references. If the alignment coverage was higher than 80% and nucleotide similarity higher than 90%, a reference-like virulence plasmid was supposed to be existing.

Statistical Analysis

The Student's t-test (for normally distributed variables) or Mann–Whitney U test (for variables that are not normally distributed) was performed to evaluate continuous variables. Dichotomous variables were analyzed by chi-square test or Fisher exact test. The associations of dichotomous and ordinal variables were evaluated by Chochran-Armitage trend test. The

strength of all associations that emerged was determined using odds ratios (ORs) and 95% confidence intervals (CIs). Two-tailed tests were used to determine statistical significance. Variables with a P-value ≤0.05 in the univariate analysis were used in cox regression for multivariate analysis to identify independent predictors. Kaplan–Meier product limit method was used to estimate the hazard ratios, and nonparametric (log rank and Wilcoxon) tests were used to compare hazard ratios in different groups. In all analyses, P-values ≤0.05 were considered significant. All statistical analyses were carried out by using the SPSS Version 23.0 (IBM Corporation, Armonk, NY, USA) and SAS v9.4 (SAS institute, Cary, NC, USA).

References

1. Friedman ND, Kaye KS, Stout JE, McGarry SA, Trivette SL, Briggs JP, et al. Health care–associated bloodstream infections in adults: a reason to change the accepted definition of community-acquired infections. Ann Intern Med. 2002;137:791–7. PubMed https://doi.org/10.7326/0003-4819-137-10-200211190-00007

2. Xiao T, Yu W, Niu T, Huang C, Xiao Y. A retrospective, comparative analysis of risk factors and outcomes in carbapenem-susceptible and carbapenem-nonsusceptible *Klebsiella pneumoniae* bloodstream infections: tigecycline significantly increases the mortality. Infect Drug Resist. 2018;11:595–606. PubMed https://doi.org/10.2147/IDR.S153246

3. McLaughlin MM, Advincula MR, Malczynski M, Barajas G, Qi C, Scheetz MH. Quantifying the clinical virulence of *Klebsiella pneumoniae* producing carbapenemase *Klebsiella pneumoniae* with a *Galleria mellonella* model and a pilot study to translate to patient outcomes. BMC Infect Dis. 2014;14:31. PubMed https://doi.org/10.1186/1471-2334-14-31

4. Qu TT, Zhou JC, Jiang Y, Shi KR, Li B, Shen P, et al. Clinical and microbiological characteristics of *Klebsiella pneumoniae* liver abscess in East China. BMC Infect Dis. 2015;15:161. PubMed https://doi.org/10.1186/s12879-015-0899-7

5. Naparstek L, Carmeli Y, Navon-Venezia S, Banin E. Biofilm formation and susceptibility to gentamicin and colistin of extremely drug-resistant KPC-producing *Klebsiella pneumoniae*. J Antimicrob Chemother. 2014;69:1027–34. PubMed https://doi.org/10.1093/jac/dkt487

6. Wang L, Shen D, Wu H, Ma Y. Resistance of hypervirulent *Klebsiella pneumoniae* to both intracellular and extracellular killing of neutrophils. PLoS One. 2017;12:e0173638. PubMed https://doi.org/10.1371/journal.pone.0173638

7. Holt KE, Wertheim H, Zadoks RN, Baker S, Whitehouse CA, Dance D, et al. Genomic analysis of diversity, population structure, virulence, and antimicrobial resistance in *Klebsiella pneumoniae*, an urgent threat to public health. Proc Natl Acad Sci U S A. 2015;112:E3574–81. PubMed https://doi.org/10.1073/pnas.1501049112

8. Bowers JR, Kitchel B, Driebe EM, MacCannell DR, Roe C, Lemmer D, et al. Genomic analysis of the emergence and rapid global dissemination of the clonal group 258 *Klebsiella pneumoniae* Pandemic. PLoS One. 2015;10:e0133727. PubMed https://doi.org/10.1371/journal.pone.0133727

9. Jiang Y, Wei Z, Wang Y, Hua X, Feng Y, Yu Y. Tracking a hospital outbreak of KPC-producing ST11 *Klebsiella pneumoniae* with whole genome sequencing. Clin Microbiol Infect. 2015;21:1001–7. PubMed https://doi.org/10.1016/j.cmi.2015.07.001

10. Moradigaravand D, Martin V, Peacock SJ, Parkhill J. Evolution and epidemiology of multidrug-resistant *Klebsiella pneumoniae* in the United Kingdom and Ireland. MBio. 2017;8:8. PubMed https://doi.org/10.1128/mBio.01976-16

11. Dong N, Zhang R, Liu L, Li R, Lin D, Chan EW, et al. Genome analysis of clinical multilocus sequence Type 11 *Klebsiella pneumoniae* from China. Microb Genom. 2018;4:54–12. PubMed https://doi.org/10.1099/mgen.0.000149

12. Gu D, Dong N, Zheng Z, Lin D, Huang M, Wang L, et al. A fatal outbreak of ST11 carbapenem-resistant hypervirulent *Klebsiella pneumoniae* in a Chinese hospital: a molecular epidemiological

study. Lancet Infect Dis. 2018;18:37–46. PubMed https://doi.org/10.1016/S1473-3099(17)30489-9

13. Li H, Durbin R. Fast and accurate short read alignment with Burrows-Wheeler transform. Bioinformatics. 2009;25:1754–60. PubMed https://doi.org/10.1093/bioinformatics/btp324

14. Croucher NJ, Page AJ, Connor TR, Delaney AJ, Keane JA, Bentley SD, et al. Rapid phylogenetic analysis of large samples of recombinant bacterial whole genome sequences using Gubbins. Nucleic Acids Res. 2015;43:e15–15. PubMed https://doi.org/10.1093/nar/gku1196

15. Stamatakis A, Hoover P, Rougemont J. A rapid bootstrap algorithm for the RAxML Web servers. Syst Biol. 2008;57:758–71. PubMed https://doi.org/10.1080/10635150802429642

16. Argimón S, Abudahab K, Goater RJE, Fedosejev A, Bhai J, Glasner C, et al. Microreact: visualizing and sharing data for genomic epidemiology and phylogeography. Microb Genom. 2016;2:e000093. PubMed https://doi.org/10.1099/mgen.0.000093

17. Didelot X, Croucher NJ, Bentley SD, Harris SR, Wilson DJ. Bayesian inference of ancestral dates on bacterial phylogenetic trees. Nucleic Acids Res. 2018;46:e134. PubMed https://doi.org/10.1093/nar/gky783

18. Yu F, Lv J, Niu S, Du H, Tang YW, Pitout JDD, et al. Multiplex PCR analysis for rapid detection of *Klebsiella pneumoniae* carbapenem-resistant (sequence type 258 (ST258) and ST11) and hypervirulent (ST23, ST65, ST86, and ST375) strains. J Clin Microbiol. 2018;56:e00731–18. PubMed https://doi.org/10.1128/JCM.00731-18

19. Diancourt L, Passet V, Verhoef J, Grimont PA, Brisse S. Multilocus sequence typing of *Klebsiella pneumoniae* nosocomial isolates. J Clin Microbiol. 2005;43:4178–82. PubMed https://doi.org/10.1128/JCM.43.8.4178-4182.2005

20. Ou W, Cui L, Li Y, Zheng B, Lv Y. Epidemiological characteristics of *bla*$_{NDM-1}$ in Enterobacteriaceae and the *Acinetobacter calcoaceticus-Acinetobacter baumannii* complex in China from 2011 to 2012. PLoS One. 2014;9:e113852. PubMed https://doi.org/10.1371/journal.pone.0113852

21. Zhou K, Ferdous M, de Boer RF, Kooistra-Smid AM, Grundmann H, Friedrich AW, et al. The mosaic genome structure and phylogeny of Shiga toxin-producing *Escherichia coli* O104:H4 is driven by short-term adaptation. Clin Microbiol Infect. 2015;21:468.e7–18. PubMed https://doi.org/10.1016/j.cmi.2014.12.009

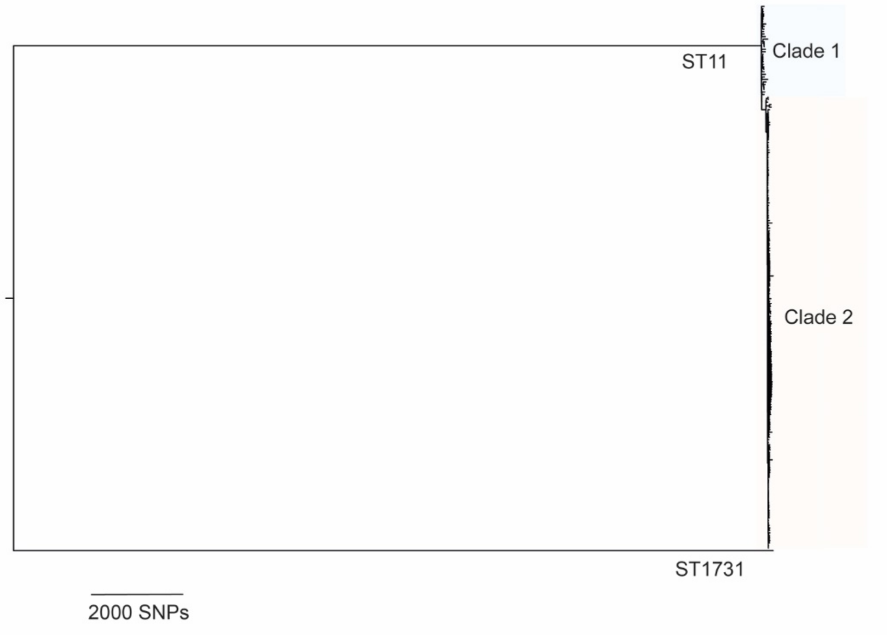

Appendix 1 Figure 1. Phylogenetic tree of 216 ST11 isolates. These include 154 CRKP isolates collected during 2012–2017 in this study, and 62 isolates that were sequenced in previous studies. The phylogenetic tree was obtained by mapping all sequence reads to the hybrid assembly of KP47434, and removing the recombined regions from the alignment. The tree was rooted using an ST1731 isolate EuSCAPE_ES29 (ERR1541319).

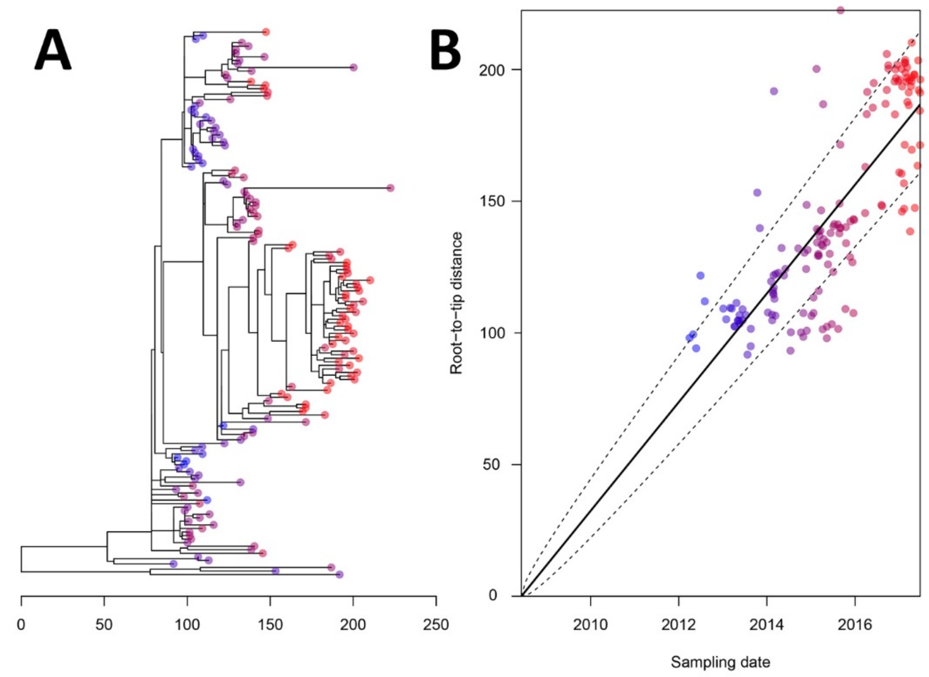

Appendix 1 Figure 2. Root-to-tip regression analyses. Plots of the root-to-tip genetic distance against sampling time are shown for the phylogeny estimated from the alignment of 154 ST11 genomes sequenced in this study. Recombinant genomic regions were excluded in this analysis.

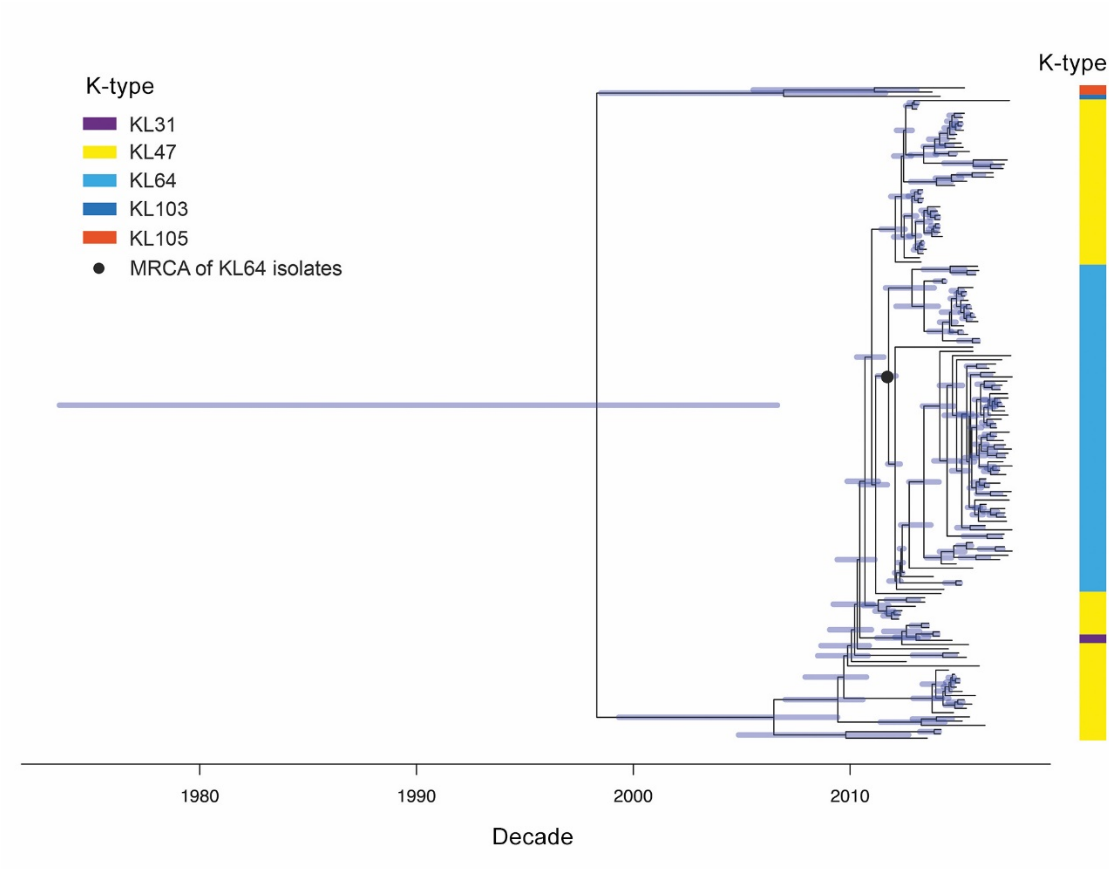

Appendix 1 Figure 3. Estimated dated phylogeny of CRKP-BSI-ST11. The tree shows the proportional relationship between branch length and time, with the bottom scale indicating time in years. The capsular type is shown on the right. Blue bars indicate 95% confidence intervals for ancestral dates.

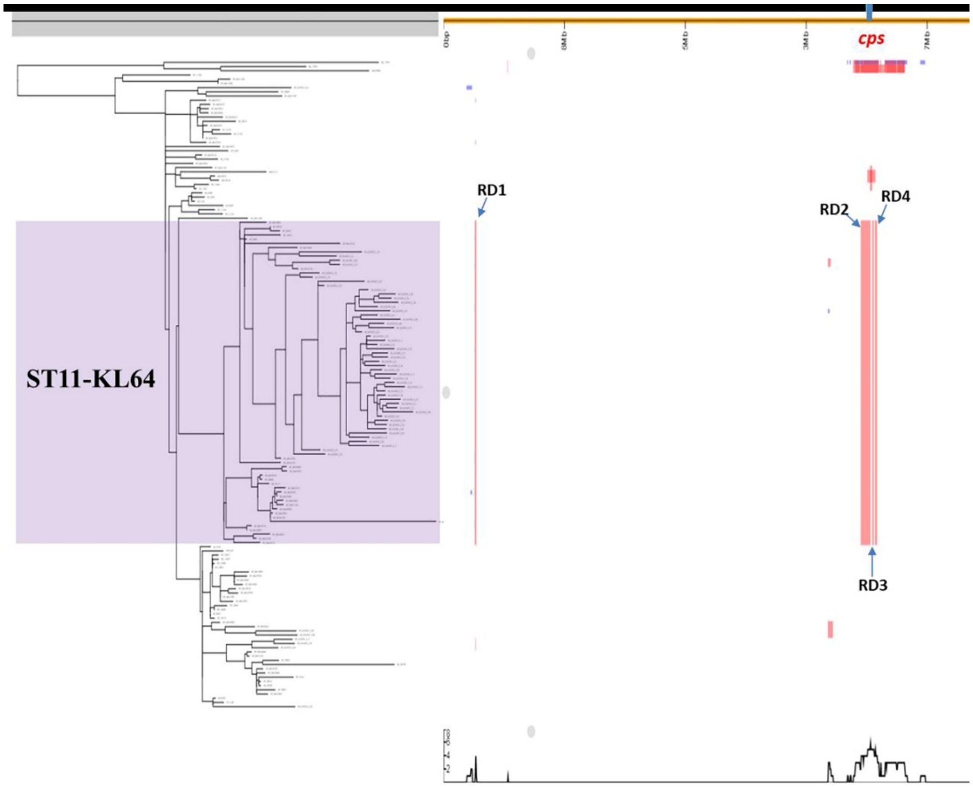

Appendix 1 Figure 4. Recombinant genomic regions detected among the 154 CRKP-ST11 isolates. Four recombinant genomic regions (RD1-RD4) were predicted by Gubbins in all CRKP-ST11-KL64 isolates highlighted in purple, and three of these were localized around the *cps* region. Very small recombinant regions cannot be seen in this figure but the details of all recombinant regions are listed in Appendix Table 3. Recombination events in the *cps* region was also predicted in isolates with other capsule types (i.e., KL31, KL103 and KL105). Figure produced with Phandango.

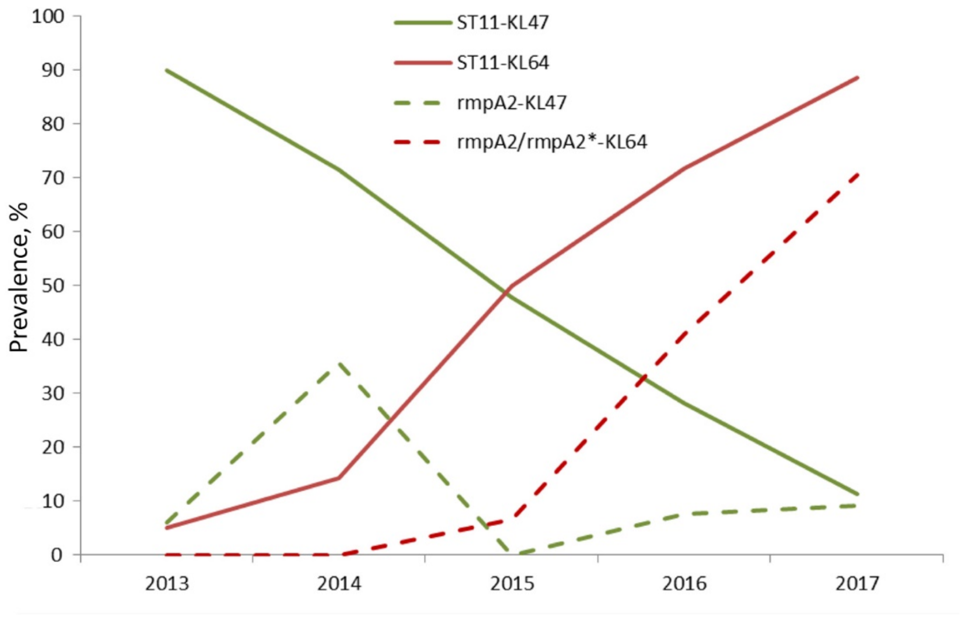

Appendix 1 Figure 5. The prevalence trend of ST11-KL47 and ST11-KL64, and *rmpA2*-KL47 and *rmpA/rmpA2**-KL64 in CRKP-ST11 population during 2013–2017 in a tertiary hospital in China.

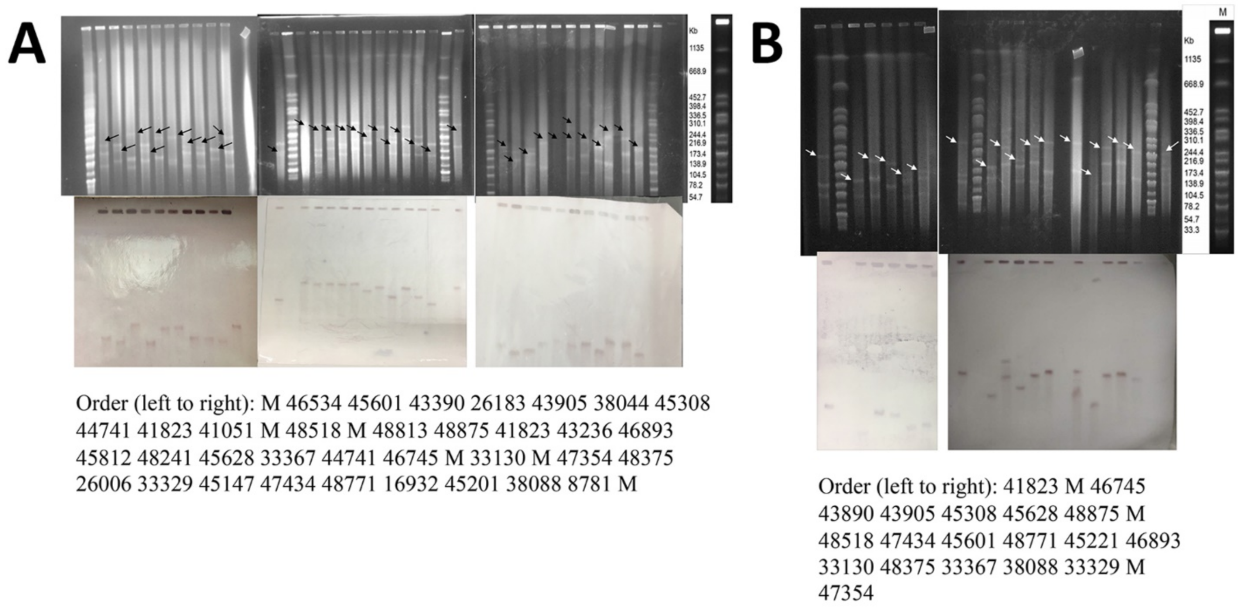

Appendix 1 Figure 6. Results of S1-PFGE and southern blotting. Representative isolates of ST11-KL47 and ST11-KL64 were selected for southern blotting to detect the location of *rmpA2* (upper) and *rmpA* (bottom). M; marker.

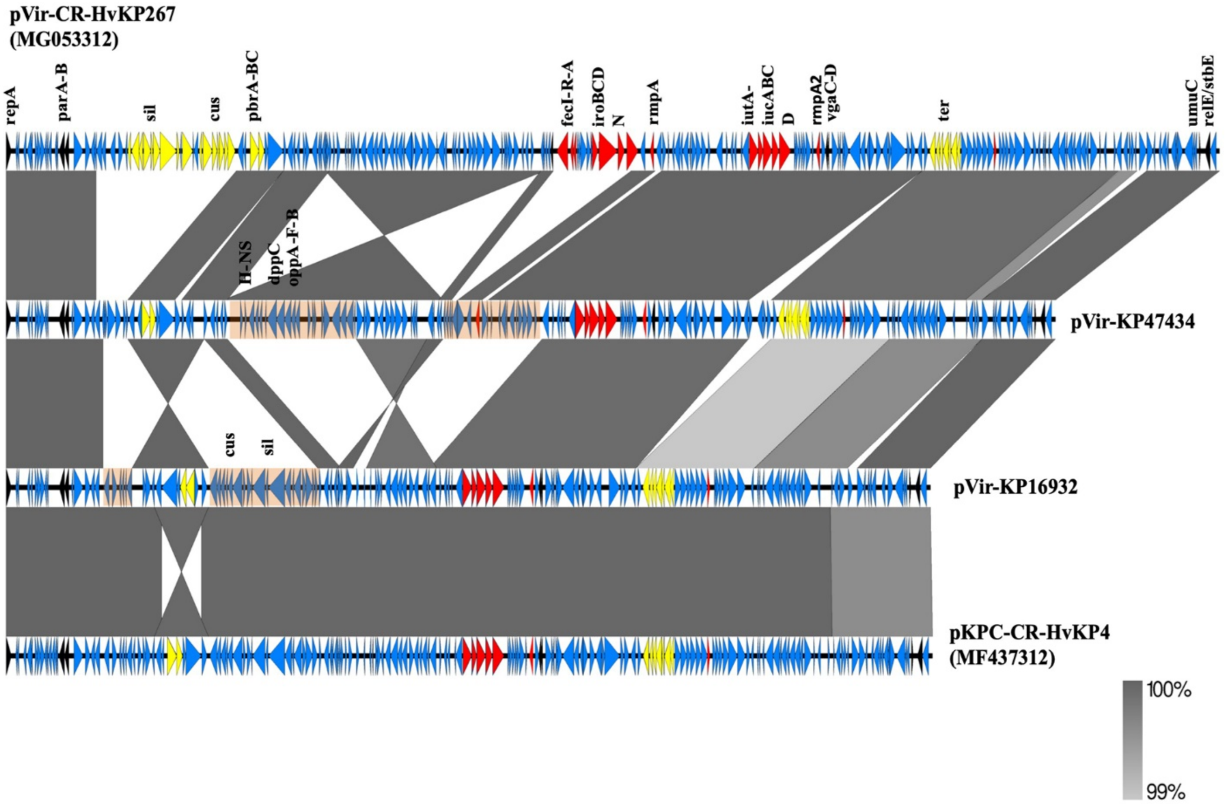

Appendix 1 Figure 7. Comparison of virulence plasmids detected in the hybrid genome assemblies of a KL47 isolate KP16932 (pVir-KP16932) and a KL64 isolate KP47434 (pVir-KP47434). Using BLASTn, we found that pKPC-CR-HvKP4 (MF437312) and pVir-CR-HvKP267 (MG053312) show the highest nucleotide similarity with pVir-KP16932 and pVir-KP47434, respectively. pKPC-CR-HvKP4 was carried by the fatal outbreak clone of hypervirulent CRKP (KL47) reported in China recently. Plasmid housekeeping genes (in black), virulence genes (in red) and resistance genes (in yellow) are indicated. Highlighted regions represent the differences detected between pVir-KP16932 and pVir-KP47434. Grey blocks represent regions of sequence homology between two plasmids.

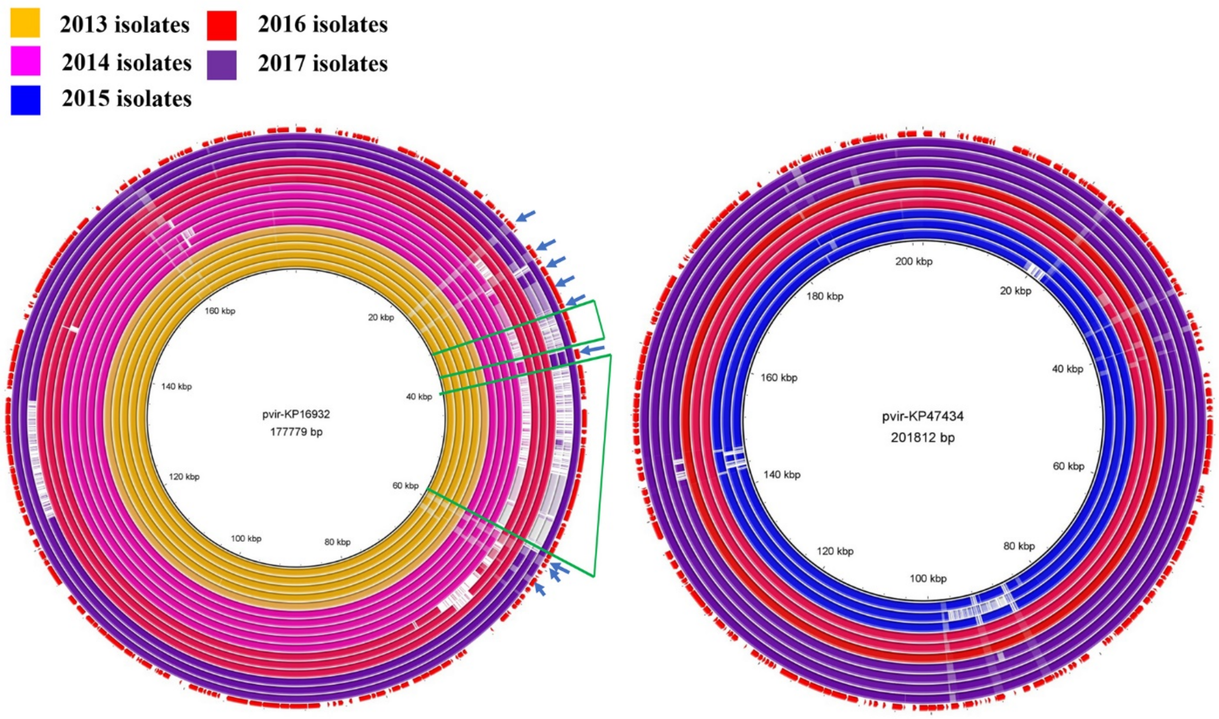

Appendix 1 Figure 8. Virulence plasmids detected in ST11-KL47 and ST11-KL64 isolates collected during 2013–2017 in this study. Plasmids pVir-KP16932 and pVir-KP47434 are as the reference to detect virulence plasmids in ST11-KL47 and ST11-KL64, respectively. Three or four isolates of each year of each subclone were randomly selected for the analysis. The contigs of each genome were blasted against the reference plasmid and plotted by BRIG. Most variations among virulence plasmids detected in ST11-KL47 were caused by gain or loss of gene clusters encoding heavy metal resistance (indicated by green frame), which are surrounded by mobile genetic elements (indicated by arrows).

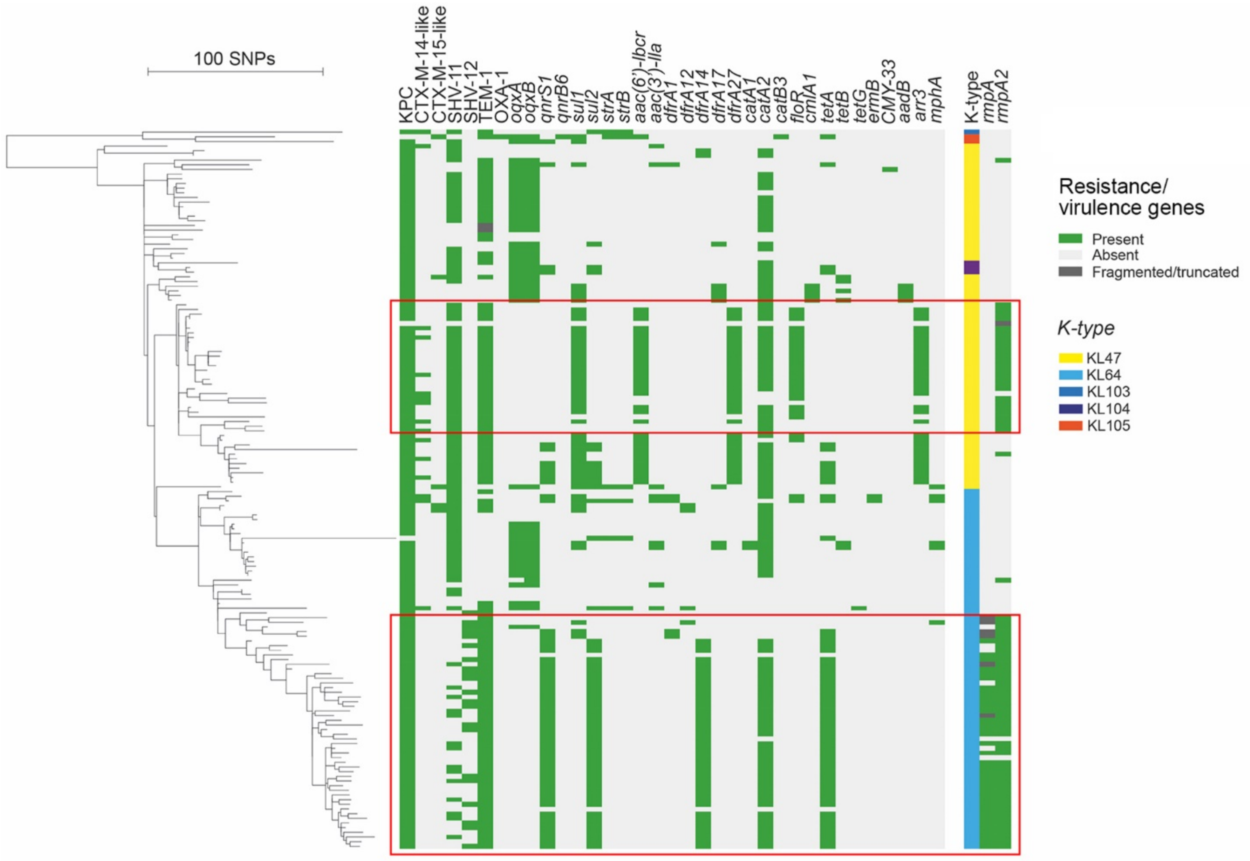

Appendix 1 Figure 9. The resistome of 154 CRKP-ST11 isolates. The resistome was determined using the program Ariba with a custom gene database.

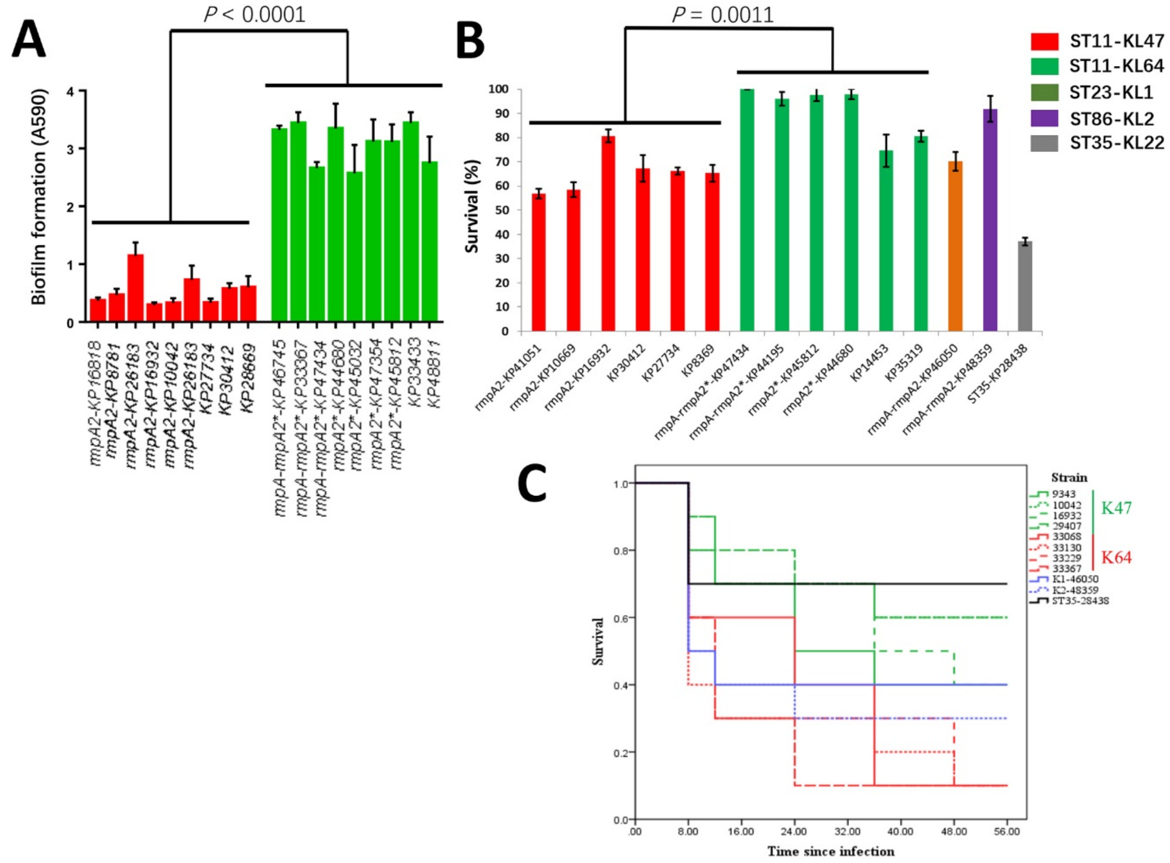

Appendix 1 Figure 10. Pathogenicity of KPC2-producing *Klebsiella pneumoniae* ST11, China, 2013–2017. The pathogenicity of *K. pneumoniae* was evaluated by assessing 3 factors. A) Biofilm production; 9 isolates of each subclone representing various genetic backgrounds were randomly selected for the assay. B) Neutrophil-killing resistance; 6 isolates of each subclone representing various genetic backgrounds were randomly selected for the human neutrophil assays. Two typical hypervirulent *K. pneumoniae* isolates (ST23-KL1 and ST86-KL2) were used as the positive control, and a classic CRKP isolate (ST35-KL22) was used as the negative control. The survival of KP47434 was set as 100%, and the survival of the other strains were recalibrated. C) Virulence potential in a *G. mellonella* infection model; 4 isolates of each subclone representing various genetic background were randomly selected for the infection assay. The survival of each strain was shown by the Kaplan–Meier curve. The positive and negative control strains were the same as those used in the human neutrophil assays. CRKP, carbapenem-resistant *Klebsiella pneumoniae;* KL, capsular loci; ST, sequence type.
